# Supplementary material for: Deletion of wfs1 Impairs Oligodendrocyte Precursor Cells Dorsal Distribution and Myelination Through the wfs1-hmgcs1 Axis in Zebrafish
Source: Biology (Basel). 2026 Aug 16;15(16):1402. doi: 10.3390/biology15161402 (PMC13510151; doi:10.3390/biology15161402)
Supplement: Supplementary file 1 [file biology-15-01402-s001.zip › File S1-Transcriptome sequencing data and differentially expressed genes (DEGs) in wfs1-- zebrafish at 5 dpf/2.KEGG/wfs1KOvsWT/UP/src/dre04210.html]

dre04210


- dre:101886621

- Up regulated genes

101886621\_1(4.59507584825526)

- dre:552927

- Up regulated genes

552927(1.53650632146374)
- dre:791531

- Up regulated genes

791531(1.26793117365426)

- dre:561021

- Up regulated genes

561021(2.14284154145004)

- dre:100000252

- Up regulated genes

100000252\_3(1.94434154854557) 100000252(1.09163460881879)

- dre:100135062

- Up regulated genes

100135062\_2(1.52157080813253)

- dre:768248

- Up regulated genes

768248(6.07490717007993)

- dre:58133

- Up regulated genes

58133(1.32041761049897)

- dre:768248

- Up regulated genes

768248(6.07490717007993)

- dre:567021

- Up regulated genes

567021(7.12614903992843)

- dre:431763

- Up regulated genes

431763(1.93865999132)

- dre:394198

- Up regulated genes

394198(1.20861445776772)
- dre:570238

- Up regulated genes

570238(2.66701017363204)

- dre:768248

- Up regulated genes

768248(6.07490717007993)

- dre:569298

- Up regulated genes

569298(1.32871616109562)

- dre:556410

- Up regulated genes

556410\_1(1.7264669285964)

- dre:57934

- Up regulated genes

57934\_1(9.91304974129429)

- dre:431777

- Up regulated genes

431777(1.35055689210441)

Close
